# Strip Surface Defect Detection Algorithm Based on YOLOv5

**DOI:** 10.3390/ma16072811

**Published:** 2023-03-31

**Authors:** Han Wang, Xiuding Yang, Bei Zhou, Zhuohao Shi, Daohua Zhan, Renbin Huang, Jian Lin, Zhiheng Wu, Danfeng Long

**Affiliations:** 1 School of Mechanical and Electrical Engineering, Guangdong University of Technology, Guangzhou 510006, China; wanghangood@126.com (H.W.); 2112101016@mail2.gdut.edu.cn (X.Y.); zhoubei0419@163.com (B.Z.); 2112201415@mail2.gdut.edu.cn (Z.S.); zhandaohua@mail2.gdut.edu.cn (D.Z.); hrenbin249@163.com (R.H.); 2112001380@mail2.gdut.edu.cn (J.L.); 2 Institute of Intelligent Manufacturing, Guangdong Academy of Sciences, Guangzhou 510070, China; 3 Guangdong Provincial Key Laboratory of Modern Control Technology, Guangzhou 510070, China

**Keywords:** deep learning, hot rolled strip steel, YOLOv5, attention mechanism, surface defect detection

## Abstract

In order to improve the detection accuracy of the surface defect detection of industrial hot rolled strip steel, the advanced technology of deep learning is applied to the surface defect detection of strip steel. In this paper, we propose a framework for strip surface defect detection based on a convolutional neural network (CNN). In particular, we propose a novel multi-scale feature fusion module (ATPF) for integrating multi-scale features and adaptively assigning weights to each feature. This module can extract semantic information at different scales more fully. At the same time, based on this module, we build a deep learning network, CG-Net, that is suitable for strip surface defect detection. The test results showed that it achieved an average accuracy of 75.9 percent (mAP50) in 6.5 giga floating-point operation (GFLOPs) and 105 frames per second (FPS). The detection accuracy improved by 6.3% over the baseline YOLOv5s. Compared with YOLOv5s, the reference quantity and calculation amount were reduced by 67% and 59.5%, respectively. At the same time, we also verify that our model exhibits good generalization performance on the NEU-CLS dataset.

## 1. Introduction

Steel surface defects have a great adverse effect on the quality of steel products. In practice, steel surface defects will cause a bad appearance, weak strength, corrosion, friction increases and other problems, causing economic losses to the forging industry. Therefore, metal surface defect detection has attracted more and more attention in recent years, and positive improvements have been made in quality control in industrial applications [1]. However, the detection of metal surface defects is easily affected by many environmental factors such as illumination, light reflection and metal materials. These factors greatly increase the difficulty of surface defect detection [2]. Therefore, it is essential for the steel industry to be able to accurately detect and discover defects in real time [3].

Since 1990, some scholars at home and abroad have been studying defect detection and classification. The main detection methods are magnetic flux leakage detection and manual visual inspection, which are time-consuming, labor-intense and expensive. In the past two decades, surface defect detection technology based on machine vision has been widely used in industrial production [4,5], gradually replacing manual detection.

According to different feature extraction methods, detection methods based on machine vision are generally divided into two categories, namely, traditional machine learning methods and deep learning methods. The former generally extract defect features through manual design parameters [6]. Commonly used handmade features include LBP (Local Binary Patterns), HOG (Histogram of Oriented Gradients), GLCM (Gray Level Co-occurrence Matrix) and other statistical features [7,8]. These detection methods are a great improvement for the detection of various surface defects. However, traditional image processing methods usually require complex threshold settings for defect recognition and are sensitive to some environmental factors such as lighting conditions and the background, so they cannot be directly applied in reality. Although researchers have developed a series of target detection models based on various strategies, artificially designed features extracted from shallow layers cannot effectively characterize images with complex backgrounds [9].

With the development of artificial intelligence and big data technology, convolutional neural networks (CNN) with powerful feature extraction ability show their unique application in surface defect detection. Tang M et al. [10] proposed a defect detection method based on an attention mechanism and multi-scale maximum pooling (MSMP). Li Z et al. [11] proposed a two-stage industrial defect detection framework based on Improved-YOLOv5 and Optimation-Inception—resnetv2, which completes the localization and classification tasks through two specific models. Liu T et al. [12] proposed an adaptive image segmentation network (AIS-Net) for the pixel-level segmentation of surface defects. In order to achieve the balance between accuracy and speed, Shi X et al. [13] proposed an improved network based on Faster R-CNN for the detection of steel surface defects. Tian R et al. [14] used key point estimation to locate the central point and regression of all other defect attributes. Secondly, an extended feature enhancement model is proposed to enlarge the receiving domain of the detector. Wang H et al. [15] proposed the first framework for the detection of defects in fewer shots. By pre-training the model using data related to the target task, the proposed framework can generate well-trained networks using a small number of labeled images. Deep learning has been successfully applied to defect classification. However, deep learning-based models still have some bottlenecks [16,17]. First, training a well-performing deep learning model relies on a large number of high-quality markup samples, but there are usually few defect samples available in practice. Second, architecture design and hyperparameter tuning are difficult. In addition, training depth models is time-consuming, especially when the architecture and hyperparameters need to be determined by trial and error [18].

Therefore, it is necessary to design a practical surface detection method with fewer parameters and a higher efficiency for practical industrial applications, which is the motivation of our research. This paper proposes a new CG-Net network model based on the YOLOv5 algorithm model, which not only exhibits better performance but also has fewer parameters and a higher efficiency. The main contributions of this paper can be summarized as follows:

- This paper proposes an ATPF (Attention Pyramid-Fast) module which can fully extract features. This module can integrate features of different scales, pay attention to a large range of location information without too much computation and extract more useful feature information.
- Based on the ATPF module, a precise and fast model framework of strip surface defect detection, CG-Net, is designed to realize the automatic, rapid and high-precision detection of strip surface defects.
- On the NEU-DET dataset, the detection average accuracy (mAP50) reaches 75.9%, mAP@0.5:0.95 reaches 39.9% and the detection speed reaches 105 frames (FPS).
- On the NEU-CLS dataset, the detection average accuracy (mAP50) reaches 59.6%, mAP@0.5:0.95 reaches 32.6% and the detection speed reaches 110 frames (FPS), which is higher than that of some advanced networks such as YOLOv5s, YOLOv3-tiny, etc.

## 2. Related Work

### 2.1. YOLOV5

**Figure 1:**
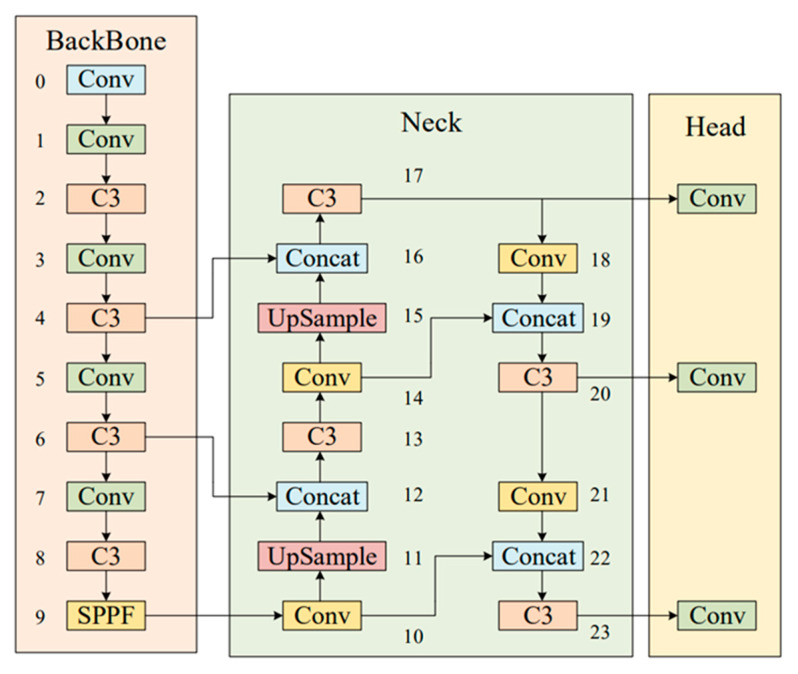
Structure diagram of YOLOv5.

The YOLO series is a representative first-level target detection technology [19]. The fifth generation of YOLO (YOLOv5) [20] was proposed in 2020 and is known as a cutting-edge object detection algorithm based on deep learning. YOLOv5 is further improved on the basis of the YOLOv4 algorithm, and the detection performance is further improved. Although the performance of the YOLOv5 algorithm was not compared and analyzed with that of the YOLOv4 algorithm, the test effect of YOLOv5 on the COCO dataset was quite good. A large number of tests are carried out on some commonly used techniques in deep learning, and some useful techniques are selected to achieve good experimental results. On the Tesla V100, the real-time detection speed of the COCO2017 dataset reaches 156 FPS, and the accuracy rate is 56.8% AP. At present, YOLOV5 is widely used in many different application scenarios, such as agriculture [21,22], industry [23,24] and other industries. In this paper, YOLOV5s is selected as the basic algorithm, taking into account the balance between the target detection accuracy and speed. The structure of YOLOv5 consists of four parts, as shown in Figure 1. The four parts are as follows:

- Input part: The input part preprocesses data training, including data preprocessing, including concatenation data enhancement [25] and adaptive image filling. To accommodate different datasets, YOLOv5 incorporates an adaptive anchor frame calculation on the input, which automatically sets the initial anchor frame size when the dataset changes.
- Main trunk: a cross-stage partial network (CSP) [26] and spatial pyramid pooling (SPPF) [27] are mainly used to extract feature graphs of different sizes from input images through multiple convolution and pooling. The bottleneck CSP is used to reduce the amount of calculation and improve the reasoning speed. The SPPF structure can realize the feature extraction of different scales from the same feature map and can generate a three-scale feature map, which is helpful in improving the detection accuracy.
- Neck: The structure combining FPN and PAN is adopted, combining the conventional FPN [28] layer with the bottom-up feature pyramid (PAN) [29] and integrating the extracted semantic features with the positional features. At the same time, the backbone layer and the detection layer are fused to make the model obtain more abundant feature information. The two structures together enhance the features extracted from different network layers in the backbone network fusion and further improve the detection capability.
- Head: The head output is mainly used to predict targets of different sizes on the feature map. YOLOV5 inherits the multi-scale prediction header of YOLOv4 and integrates three-layer feature mapping to improve the detection performance of different target sizes.

### 2.2. Lightweight Network

In order to find the best balance between computational cost and detection efficiency, the researchers explored different methods for reducing the scale and computational cost of neural networks. Some studies focus on reducing the bit accuracy of weights to make the model more compact [30]. Other works are based on the distillation of knowledge [31], which dissolves large architectures into smaller ones. In addition, more attempts have been made to reduce the number of less influential parameters in the pruning training model [32,33].

Lan R et al. [34] proposed a dense lightweight network, called MADNet, for stronger multi-scale feature expression and feature correlation learning. Shin Y G [35] proposed a new parallel extended decoder path semantic patching network structure to reduce hardware costs and improve semantic patching performance. Zhou Q et al. [36] designed a lightweight encoder–decoder network for the real-time semantic segmentation of autonomous driving images. Liu C et al. [37] constructed a network with extended convolution and attention modules as the backbone network for feature extraction and used pooling operations of different sizes to encode the surrounding semantic information on the extended pyramid pooling module ASPP. Liang H et al. [38] proposed a lightweight end-to-end road damage detection network, which can quickly, automatically and accurately identify and classify various types of road damage.

However, these methods are often achieved by compressing pre-trained networks or directly training small networks that pay close attention to model size rather than their overall performance. On the premise of considering the performance, the network proposed in this paper effectively reduces the amount of computation and the scale of the model and truly realizes the lightweight and high efficiency.

## 3. Method

**Figure 2:**
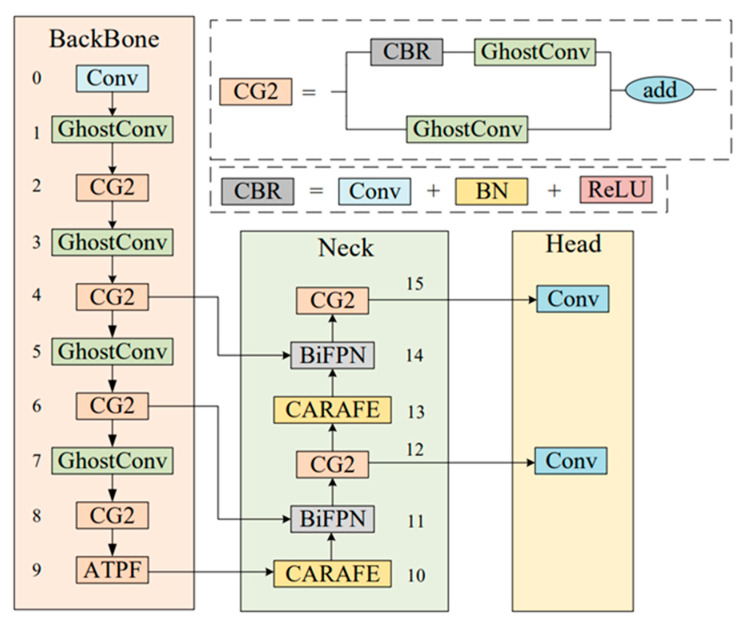
Network structure diagram of CG-Net.

The YOLOV5 network with C3 as the backbone can recognize more complex features. Therefore, based on the structure of C3, the CG2 module is proposed in this paper. At the same time, a new feature fusion method, ATPF, is proposed, which can carry out adaptive weighting according to the contribution to the space and channel so that the network is more sensitive to useful channels or spatial information and can improve the multi-scale recognition ability of the network to chip defects. Since the training calculation and reasoning speed cost of the YOLOv5s model is much lower than that of the other four models, in order to pursue the balance between detection speed and accuracy, we choose to use YOLOv5s as our identification network for improvement. Therefore, based on the network structure of YOLOv5s combined with the CG2 module and ATPF module, a network, CG-Net, for strip surface defect detection is proposed in this paper. The network structure is shown in Figure 2. Next, the CG2 module and ATPF module proposed in this paper will be introduced in detail.

### 3.1. CG2 Module

The structure of the C3 module in Yolov5 is a bottleneck composed of three general convolutions and a bottleneck, while the CG2 module changes its bottleneck to GhostConv on the basis of C3 and its general convolution on the branch to GhostConv. In order to solve the problems caused by an overly deep network depth, such as gradient disappearance, gradient explosion and overfitting, the Concat operation is changed to residual connection, and the last common convolution is removed.

**Figure 3:**
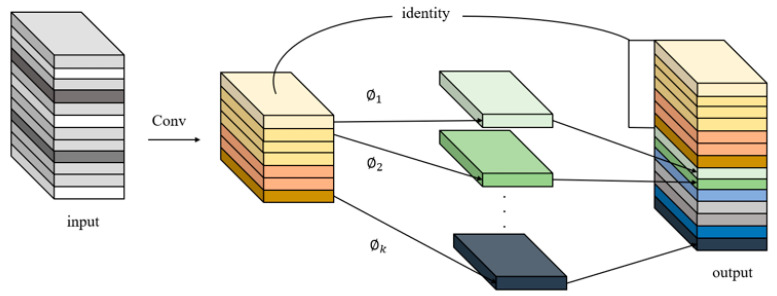
GhostConv structure diagram.

Deep neural networks generate many similar redundant feature maps when extracting features. Although they are important for deep neural networks to understand data characteristics, generating them in convolution operations requires a lot of computation. Inspired by GhostNet [39], GhostNet is a neural architecture designed to verify the effectiveness of GhostConv. We introduced GhostConv in the process of feature space expansion to generate more feature graphs from cheap operations, thus reducing the memory consumption in the process of intermediate expansion. At the same time, in order to ensure the effective extraction of our feature information and improve the stability of our network, we introduce residual connection into the CG2 module. At the same time, in order to ensure the effective extraction of our feature information and improve the stability of our network, we introduce residual connection into the CG2 module. The structure of GhostConv is shown in Figure 3:

The residual connection can effectively solve a series of problems caused by the increase in the network depth, such as gradient disappearance, gradient explosion and the easy overfitting of the model. We added residual connection in the CG2 module to avoid the overfitting problem caused by the increase in network layers so as to effectively improve the stability of our network. The input and output of the first layer are defined as *x* and *y* respectively, and the nonlinear change in the input is defined as $F\left(x,\left\{W_{i}\right\}\right)$. Then, the formula for calculating residual connection is as follows:

(1) $$y=F\left(x,\left\{W_{i}\right\}\right)+x,$$

The introduction of the GhostConv module and residual structure in the CG2 module can greatly reduce the amount of computation and obtain enough feature graphs to ensure the stability of the network.

### 3.2. ATPF Module

**Figure 4:**
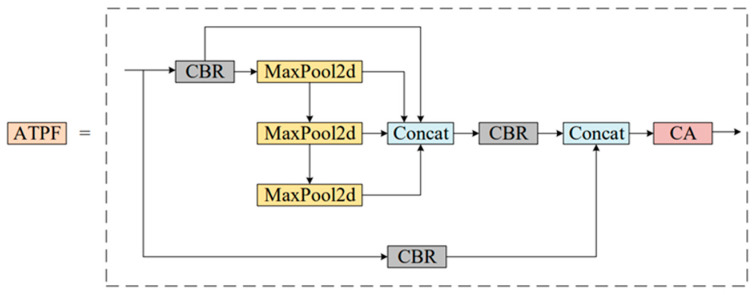
Structure diagram of the ATPF module.

In order to make better use of different scale features, this paper proposes a new spatial scale fusion module (ATPF), whose structure is shown in Figure 4. The ATPF consists of spatial scale fusion and attention modules, and the feature map is processed by these two blocks in turn. Spatial scale fusion usually adopts SPPF, which focuses on spatial information and consists of four parallel branch connections: three maximum pooling operations (kernel size 5 × 5, 9 × 9, 13 × 13) and the input itself. After a convolution operation of the input features, three maximum pooling operations (convolution kernel size is 5 × 5, 9 × 9, 13 × 13) are adopted, respectively, to receive the feature information of different scales. Then, the feature graphs after convolution and maximum pooling are superimposed on the dimension of the channel to ensure that the feature information is not lost. Then, the number of 4c (channels) is reduced to c by 1 × 1 convolution. At the same time, after the input is convolved with another line, it is spliced again with the output after the dimension reduction in the channel. The spliced feature chart shows the number of channels (2c). Finally, the CA attention mechanism module is introduced in the series. Again, the number of channels is converted from 2c to c.

The spatial scale fusion part of the ATPF module uses the SPPF module, and the other part is the attention mechanism module. The attention weighting block is an adaptive regulator whose function is to learn the importance of the spatial information of each channel, to save resources by focusing limited attention on the key information and, thus, to show which scale features are more significant. Although multi-scale information is the basis of effective feature maps, different scales contribute different results. Therefore, the attention weighting block adaptively assigns weight to different scales in the process of network learning. The more significant the information, that is, the more meaningful the scale features, the more weight they assign.

Currently, the commonly used attention mechanisms include the SE, CBAM, ECA and CA modules, among which SE is to increase the attention mechanism in the channel dimension. This module obtains the importance of each channel in the feature graph through automatic learning and uses the importance obtained to improve the features and suppress the features that are not important to the current task. CBAM automatically acquires the importance of each feature channel through learning, similar to SE. In addition, the importance of each feature space is automatically obtained through a similar learning method. The importance is used to promote features and suppress features that are not important to the task at hand. The ECA module avoids dimension reduction and effectively captures cross-channel interactions. The module only adds a few parameters but can obtain an obvious performance gain. The CA module can encode the horizontal and vertical location information into the pass so that the mobile network can pay attention to a large range of location information without too much computation.

In general, the proposed ATPF module improves the context representation ability of feature graphs by integrating more information sources and adaptively weighting them according to their importance.

### 3.3. CARAFE

**Figure 5:**
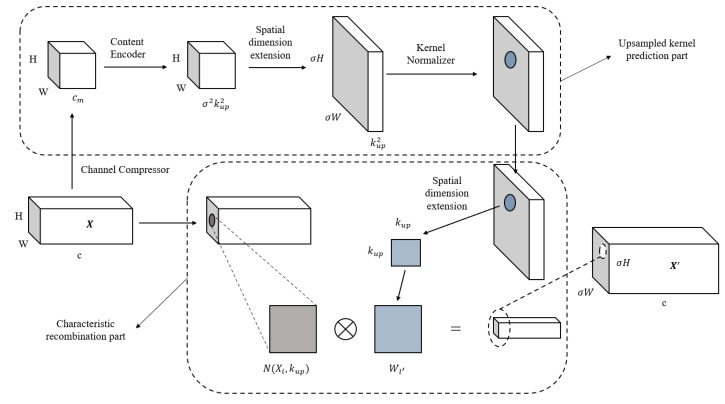
Structure diagram of the CARAFE up-sampling operator.

Feature up-sampling is a key operation of many modern convolutional network architectures developed for tasks such as object detection, instance segmentation and scene resolution. There are two main up-sampling methods used. One is the linear difference method: the nearest neighbor difference algorithm and bilinear difference, which mainly focus on subpixel space and cannot capture rich semantic information. The other is deconvolution, which achieves dimension expansion through convolution. However, deconvolution uses the same convolution kernel for the whole image, which limits the perception ability of local changes, and it cannot have a good response ability to local changes. It also increases the number of parameters. Wang et al. proposed a CARAFE [40] up-sampling operator. In this paper, we use content-aware feature recombination (CARAFE) to sample the feature map. At each location, CARAFE can use the underlying content information to predict the reassembled kernel and reassemble features within a predefined neighborhood. The CARAFE up-sampler has made remarkable progress with only a few extra parameters and computation work. Because of the content information, CARAFE can use adaptive and optimized reassembled kernels in different locations and achieve better performance than mainstream up-sampling operators such as interpolation or deconvolution. The network structure of CARAFE is shown in Figure 5:

### 3.4. BiFPN

**Figure 6:**
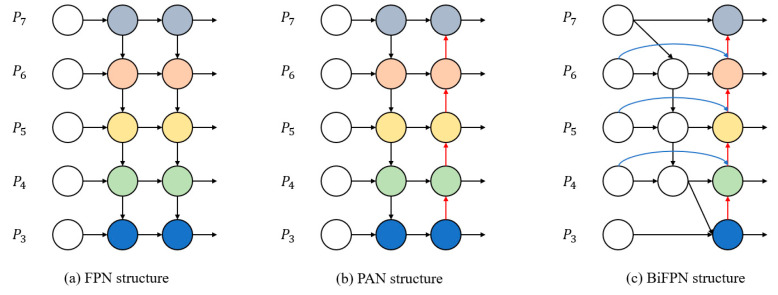
Structure of FPN, PAN and BiFPN [41].

In the YOLOv5 algorithm, the FPN+PAN structure is used in the neck part, which achieves good results in multi-scale fusion. However, its calculation is complicated, the current task image is easily affected by environmental factors and the scale is diverse, so the structure has insufficient feature extraction and utilization, resulting in large loss errors. Therefore, the bidirectional feature fusion structure BiFPN [41] is introduced in the neck part, and the BiFPN structure is shown in Figure 6.

The BiFPN structure is based on PAN. Compared with the original neck structure, BiFPN removes nodes without feature fusion and contributes little, and it adds new channels between input nodes and output nodes at the same level, thus combining more feature information while saving resource consumption. At the same time, a cross-scale connection method is proposed, and an extra edge is added to integrate the features in the feature extraction network directly with the features relative to the size in the bottom-up path so that the network can retain more superficial semantic information without losing too much relatively deep semantic information. BiFPN enhances the information extraction capability of the network so that the low-level location information can be combined with the high-level semantic information, which further improves the target detection performance of the network.

## 4. Experimental Simulation and Analysis

In order to demonstrate the superiority of the frame in the surface defect identification of hot rolled steel strip, experimental results and analysis are given in this section. In this section, we first introduce datasets, experimental parameter settings and evaluation metrics. Ablation studies then confirmed the contribution of the GhostConv, CG2, ATPF and CARAFE and BiFPN modules. Specifically, the ablation study was designed to demonstrate the necessity and to visualize the weight values to demonstrate the weight allocation mechanism described above. Finally, the proposed method is compared with other advanced methods for the task of defect identification.

### 4.1. Dataset

#### 4.1.1. NEU-DET Dataset

**Figure 7:**
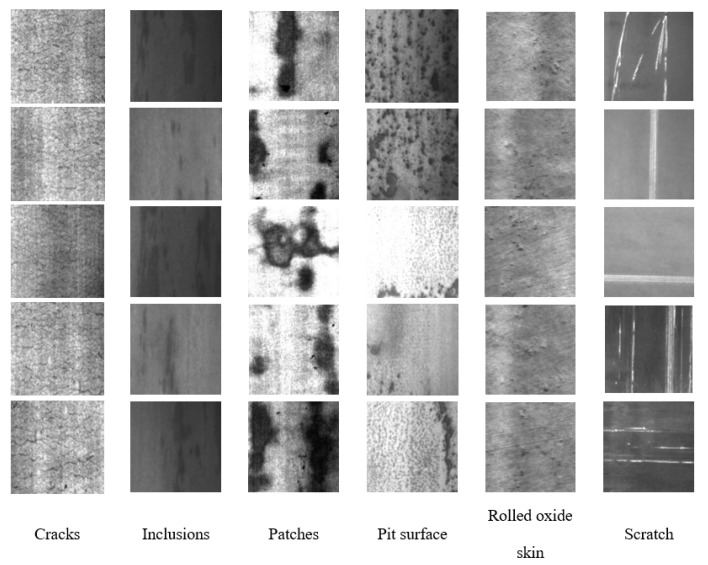
Partial defect samples of the NEU-DET dataset.

In order to verify the effectiveness of the proposed method, the public dataset NEU-DET [42] was introduced in our experiment to evaluate the performance of CG-Net and some recent models. There are six defect types in the NEU-DET dataset: scratches, patches, pitted surface, inclusion, crazing and rolled oxide scale. Each defect type has 300 images with a resolution of 200 by 200 pixels. There are 1800 grayscale images in total. The NEU-DET dataset was divided into a training set and a test set in a ratio of 90% and 10%, so 1620 samples were used for training and 180 samples were used for testing. The training set is used to train network parameters to minimize the loss function. The test set was used to evaluate the accuracy of the trained network in identifying surface defects. Figure 7 shows samples of six typical surface defects.

#### 4.1.2. NEU-CLS Dataset

The NEU surface defect (NEU-CLS) dataset published by Song et al. [42] was mainly used in our experiments to evaluate the performance of CG-Net and some state-of-art models. The NEU-CLS dataset contains six types of defects in total, i.e., scratch (Sc), patch (Pa), pitted surface (Ps), inclusion (In), crazing (Cr) and rolled-in scale (Rs). Each defect type has 300 images with a resolution of 200 × 200 pixels. A total of 1800 grayscale images are present. The NEU-CLS dataset was divided into a training set and a test set in a ratio of 90% and 10%, so 1620 samples were used for training and 180 samples were used for testing.

### 4.2. Experimental Parameter Setting

This experiment was carried out on the PyTorch deep learning framework. This experiment used an NVIDIA GeForce RTX 3090 graphics card with 24 gigabytes of video memory and an Interl 3.00 GHz i9-10980XE CPU. The network training process consisted of 150 epochs. The random gradient (SGD) descent optimizer was used, the batch size was 8 and the linear attenuation learning rate scheduling strategy was adopted, with an initial learning rate of 0.01 and a final learning rate of 0.0001. The momentum parameters and weight attenuation are 0.937 and 0.0005, respectively. The input image was uniformly transformed to a size of 640 × 640 and normalized.

### 4.3. Evaluation Index

The mean average precision (mAP), Recall (Recall), FLOPs (floating point operation), Params (parameters) and frames per second (FPS) were used to comprehensively evaluate the proposed network. In the task of the surface defect detection of hot rolled strip steel, the intersection ratio (IOU) is used to judge whether the detected result is a true defect. If the value exceeds the threshold set, it is considered a positive sample; otherwise, it is a negative sample. In the target detection task, the accuracy and recall rate are important indicators in judging the recognition effect of the network, which are defined as follows:

(2) $$\mathrm{Precision}=\mathrm{TP}/\left(\mathrm{TP}+\mathrm{FP}\right),$$

(3) $$\mathrm{Recall}=\mathrm{TP}/\left(\mathrm{TP}+\mathrm{FN}\right),$$

TP, FP and FN are explained as follows:

- true positive (TP): it means the correct prediction was right in the case of the sample;
- false positive (FP): it means the error prediction was right in the case of the sample;
- false negative (FN): it means the sample error prediction for a negative example.

mAP50:95 represents the average mAP at different IoU thresholds (from 0.5 to 0.95, step size 0.05) (0.5, 0.55, 0.6, 0.65, 0.7, 0.75, 0.8, 0.85, 0.9, 0.95). It better represents the performance of the model. Therefore, MAP50:95 was used to replace mAP50 in evaluating the performance of our model. In addition, in order to compare the computational complexity of different networks, we chose the computational time complexity (FLOPs) and computational space complexity Params (parameter number) to represent the differences between different methods. In addition, during the test phase, FPS was used to represent the reasoning speed, and the result of FPS was the average of 180 test images.

### 4.4. Ablation Experiment

We used ablation experiments to verify the advantages of the GhostConv, CG2, ATPF and CARAFE and BiFPN modules in CG-Net networks. The experimental results are shown in Table 1 and Table 2 below. GhostConv, CG2, ATPF and CARAFE and BiFPN can improve the detection speed while improving the accuracy and reducing the number of parameters and the calculation amount, but they are not compatible with the detection accuracy, the number of parameters, the calculation amount and the detection speed. After the introduction of five modules in experiment 6, the detection accuracy is 4% higher than that in experiment 1, the number of parameters and the calculation amount are reduced by 67% and 59.5%, respectively, and the detection speed is also increased by nine frames. Similarly, in the NEU-CLS dataset, the experimental results of experiment 6 are significantly better than those of other experiments. Under the premise of considering the performance, the calculation amount and model scale are effectively reduced, and the lightweight and high efficiency are truly realized. In order to detect the surface defects of hot rolled strip steel in real time and accurately, the combination of experiment 6 is more in line with the requirements.

The ATPF module introduces the attention mechanism. Currently, the four commonly used modules of the attention mechanism are SE, CA, CBAM and ECA. As shown in Table 3 and Table 4, after four different attention mechanism modules are introduced into the ATPF module, the CA detection result is the highest among the four, so we pay more attention to the improvement of accuracy. Therefore, we adopt CA as the attention module in ATPF.

### 4.5. Advanced Model Comparison

To verify the strip surface defect detection performance of our CG-Net, we compared our approach to a number of recent models, including the networks YOLOv3, Yolov3-tiny, YOLOv5s and YOLOv7-tiny. In addition, we replaced the default backbone of YOLOv5s with the lightweight backbone MobileNetV3, ShuffleNetv2 and GhostNet. Table 5 shows the results of the quantitative comparison of each network on the NEU-DET dataset. Our CG-Net method achieves 39.9% mAP, which is superior to all other methods, and its complexity is significantly lower than that of all classical network models at only 2.3 M Params and 6.5 GFLOPs. The YOLOv3-tiny has the highest FPS, but its detection performance is unsatisfactory, with only 22.4% of the mAP. Our CG-Net has achieved the best results in terms of the three aspects of detection accuracy, parameter number and computation amount, and its detection performance is better than that of all lightweight networks and most first-level networks. We improved the detection speed by 9FPS compared to the baseline YOLOv5s and reduced the number of parameters and calculations by 67% and 59.5% compared to YOLOv5s, respectively, and it was 2.6 times faster than YOLOv3. Meanwhile, the detection speeds of MobileNetv3-YOLOv5, ShuffleNetv2-YOLOv5 and GhostNetS-YOLOv5, which replaced the backbone, were all lower than that of the baseline YOLOv5s.

Table 6 shows the results of the quantitative comparison of each network on the NEU-CLS dataset. It can be seen in the table that our network has achieved the optimal comprehensive performance with the fewest number of parameters and the least amount of computation.

**Figure 8:**
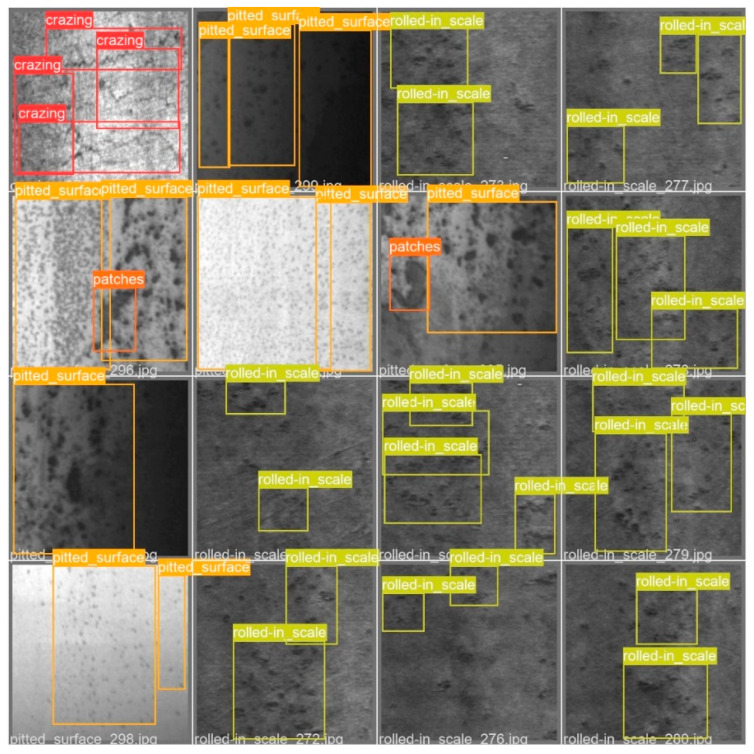
Detection results of defects in the NEU-DET dataset by the proposed algorithm.

The test results of CG-Net are shown in Figure 8. It can be seen that our CG-Net is capable of processing strip surface defect images under various types and lighting conditions.

## 5. Conclusions

In this paper, CG-Net, a lightweight defect detection method based on YOLOv5, is designed. The CG2 module and ATPF module are designed for six defects of hot rolled strip steel. The BiFPN structure was adopted to improve the ability of the detector to adjust objects of different scales through the fusion of different scale characteristics. Second, this paper proposes using the CARAFE module to replace bilinear interpolation up-sampling. The CARAFE module can increase the receptive field of up-sampling and is based on content up-sampling, so it can extract more image features and improve model performance. Through testing on the NEU-DET dataset, CG-Net achieved 75.9% mAP at only a 2.3 MB model size and 6.5 GFLOPs, an improvement of 6.3 points over YOLO v5s, with an FPS of 105. Compared with YOLO v5s, the reference quantity and calculation amount are reduced by 67% and 59.5%, respectively. At the same time, we also verify that our model has good generalization performance on the NEU-CLS dataset. In the future, we will focus on the further optimization of the algorithm to achieve a higher accuracy, faster detection speed and lower model complexity.

## Figures and Tables

**Table 1:** Ablation experiments of CG-Net (NEU-DET dataset).

Number	GhostConv	CG2	ATPF	CARAFE + BiFPN	mAP50	mAP50:95	Params(M)	GFLOPs(G)	FPS
1	—	—	—	—	69.6	35.9	7.0	15.8	96
2	√	—	—	—	74.1	38.0	3.9	11.4	120
3	—	√	—	—	71.5	37.1	2.9	7.7	147
4	—	—	√	—	72.2	38.6	4.6	13.1	114
5	—	—	—	√	72.4	37.9	4.9	13.8	103
6	√	√	√	√	75.9	39.9	2.3	6.5	105

**Table 2:** Ablation experiments of CG-Net (NEU-CLS dataset).

Number	GhostConv	CG2	ATPF	CARAFE + BiFPN	mAP50	mAP50:95	Params(M)	GFLOPs(G)	FPS
1	—	—	—	—	57.6	30.6	7.0	15.8	99
2	√	—	—	—	58.6	31	3.9	11.4	120
3	—	√	—	—	58.4	30.8	2.9	7.7	145
4	—	—	√	—	60.2	32.1	4.6	13.1	113
5	—	—	—	√	57.9	30.8	4.9	13.8	103
6	√	√	√	√	60.8	32.6	2.3	6.5	110

**Table 3:** Comparison of effects of different attention mechanisms in the ATPF module (NEU-DET dataset).

SE	CA	CBAM	ECA	P	R	mAP50	mAP50:95
√	—	—	—	66.4	67.8	73.5	39.0
—	√	—	—	73.4	68.7	75.9	39.9
—	—	√	—	71.4	68	73	39.5
—	—	—	√	67.9	68.2	73.1	38.6

**Table 4:** Comparison of effects of different attention mechanisms in the ATPF module (NEU-CLS dataset).

SE	CA	CBAM	ECA	P	R	mAP50	mAP50:95
√	—	—	—	59.4	63.1	61.1	31.9
—	√	—	—	54.3	64.2	60.8	32.6
—	—	√	—	58.9	62.2	59.6	32.5
—	—	—	√	54.8	62.1	58	30.9

**Table 5:** Comparison with state-of-the-art methods on the NEU-DET dataset.

Method	mAP50	mAP50:95	Params(M)	GFLOPs(G)	FPS
YOLOv3	73.1	37.0	61.5	154.6	40
YOLOv3-tiny	54	22.4	8.6	12.9	**172**
YOLOv5-s	69.6	35.9	7.0	15.8	96
MobileNetv3-YOLOv5	71.9	36.6	5.0	11.3	72
ShuffleNetv2-YOLOv5	63.7	31.5	3.8	8.0	83
GhostNet-YOLOv5	73.2	36.6	4.7	7.6	74
YOLOv7-tiny	69.3	32.6	6.0	13.1	99
CG-Net	**75.9**	**39.9**	**2.3**	**6.5**	105

**Table 6:** Comparison with state-of-the-art methods on the NEU-CLS dataset.

Method	mAP50	mAP50:95	Params(M)	GFLOPs(G)	FPS
YOLOv3	60.1	28.4	61.5	154.6	39
YOLOv3-tiny	38.9	14.1	8.6	12.9	**222**
YOLOv5-s	57.6	30.6	7.0	15.8	99
MobileNetv3-YOLOv5	57.6	29.1	5.0	11.3	76
ShuffleNetv2-YOLOv5	53.9	24.5	3.8	8.0	83
GhostNet-YOLOv5	58.9	30.9	4.7	7.6	56
YOLOv7-tiny	54	26.1	6.0	13.1	101
CG-Net	**60.8**	**32.6**	**2.3**	**6.5**	110

## Data Availability

The raw/processed data and modeling codes required to reproduce these findings cannot be shared at this time, as the data also form part of an ongoing study.
